# The Effect of Ovariectomy and Estradiol Substitution on the Metabolic Parameters and Transcriptomic Profile of Adipose Tissue in a Prediabetic Model

**DOI:** 10.3390/antiox13060627

**Published:** 2024-05-21

**Authors:** Irena Marková, Martina Hüttl, Denisa Miklánková, Lucie Šedová, Ondřej Šeda, Hana Malínská

**Affiliations:** 1 Centre for Experimental Medicine, Institute for Clinical and Experimental Medicine, 140 21 Prague, Czech Republic; mabw@ikem.cz (M.H.); mild@ikem.cz (D.M.); haml@ikem.cz (H.M.); 2 Institute of Biology and Medical Genetics, First Faculty of Medicine, Charles University and General University Hospital, 121 08 Prague, Czech Republic; lsedo@lf1.cuni.cz (L.Š.); oseda@lf1.cuni.cz (O.Š.)

**Keywords:** ovariectomy, estradiol substitution, perimetrial adipose tissue, transcriptomics, hereditary hypertriglyceridemic rat, insulin sensitivity

## Abstract

Menopause brings about profound physiological changes, including the acceleration of insulin resistance and other abnormalities, in which adipose tissue can play a significant role. This study analyzed the effect of ovariectomy and estradiol substitution on the metabolic parameters and transcriptomic profile of adipose tissue in prediabetic females of hereditary hypertriglyceridemic rats (HHTgs). The HHTgs underwent ovariectomy (OVX) or sham surgery (SHAM), and half of the OVX group received 17β-estradiol (OVX+E2) post-surgery. Ovariectomy resulted in weight gain, an impaired glucose tolerance, ectopic triglyceride (TG) deposition, and insulin resistance exemplified by impaired glycogenesis and lipogenesis. Estradiol alleviated some of the disorders associated with ovariectomy; in particular, it improved insulin sensitivity and reduced TG deposition. A transcriptomic analysis of perimetrial adipose tissue revealed 809 differentially expressed transcripts in the OVX vs. SHAM groups, mostly pertaining to the regulation of lipid and glucose metabolism, and oxidative stress. Estradiol substitution affected 1049 transcripts with overrepresentation in the signaling pathways of lipid metabolism. The principal component and hierarchical clustering analyses of transcriptome shifts corroborated the metabolic data, showing a closer resemblance between the OVX+E2 and SHAM groups compared to the OVX group. Changes in the adipose tissue transcriptome may contribute to metabolic abnormalities accompanying ovariectomy-induced menopause in HHTg females. Estradiol substitution may partially mitigate some of these disorders.

## 1. Introduction

Menopause is the permanent cessation of menstruation resulting from estrogen deficiency, which occurs naturally as women age. This phase is pivotal, not only in terms of reproductive health, but also in its far-reaching metabolic implications. It is being increasingly recognized that menopause is associated with weight gain, the development of visceral obesity, insulin resistance, and other disorders of the lipid and carbohydrate metabolism. These changes significantly increase the risk of developing metabolic syndrome (MS), type 2 diabetes, and cardiovascular disease (CVD) [1,2,3]. The risk of developing MS in women after menopause is 60% compared to 20–30% in age-matched premenopausal women [4]. Additionally, up to 80% of patients with MS die due to MS cardiovascular complications [5].

Adipose tissue plays a key role in the development of metabolic changes after menopause. Estrogens are responsible for sex-specific differences in fat distribution and accumulation and play an important role in the regulation of energy homeostasis, body weight, and glucose and lipid metabolism [6,7]. Ovarian hormones regulate the immune response and inflammation and contribute to the maintenance of insulin sensitivity in white adipose tissue. Their deficiency after menopause is accompanied by a redistribution of fat reserves from the subcutaneous to visceral stores, elevating the risk of metabolic disease [8]. It has been shown that estrogens administered to ovariectomized mice normalized body weight and restored insulin sensitivity [9]. A study using ovariectomized rats showed that 17β-estradiol replacement suppressed body weight gain and fat accumulation, enhancing whole-body energy consumption not only under a standard diet [10], but also under a high-fat diet [11]. These studies indicate that estradiol affects insulin sensitivity and confirm the important role of estrogens in the regulation of energy balance.

In women without diabetes, hormone replacement therapy reduced abdominal obesity, insulin resistance, blood pressure, and new-onset diabetes and decelerated atherosclerosis [12], while in diabetic women, hormone therapy decreased fasting glucose and improved insulin resistance [13]. A higher prevalence of metabolic disorders after menopause indicates that the disruption of ovarian function may contribute to the occurrence of these conditions. Although the efficacy of hormone therapy for the management of menopause symptoms is well established, its relationship with cardiovascular outcomes is rather complex [14], and hormone therapy is not currently recommended for the prevention of diabetes [15].

In our previous study on ovariectomized Wistar female rats, we found that hepatic lipid dysmetabolism plays an important role in the early events of postmenopausal metabolic syndrome, and reduced lipogenesis in white and brown adipose tissue may contribute to the development of insulin resistance in peripheral tissues [16]. Boldarine et al. elucidated a trio of metabolic disturbances in the retroperitoneal adipose tissue of ovariectomized rats: a marked impairment in fatty acid oxidation, a heightened proinflammatory state, and an increase in saturated fatty acids content [17]. These findings suggest the visceral adipose tissue as a site at which major changes occur after ovariectomy and which is fundamentally involved in the development of changes after menopause. Therefore, it is important to identify the changes that underlie the development of disorders associated with the ovariectomy/postmenopausal period.

In the current study, we aimed to investigate the effect of ovariectomy and estradiol substitution on the metabolic parameters, insulin sensitivity, and transcriptomic profile of adipose tissue in a prediabetic model with insulin resistance. We used a non-obese strain of hereditary hypertriglyceridemic rats (HHTg), an established model of metabolic syndrome which exhibits insulin resistance, dyslipidemia, liver steatosis, impaired glucose tolerance, and increased oxidative stress [18,19,20]. We focused on the perimetrial adipose tissue of female rats, attributing its heightened sensitivity compared to the other depots to the effects of sex hormones [21] and its association with impaired health, increased cardiometabolic risk, and proneness to inflammation [22]. We studied the metabolic changes and shifts in global gene expression caused by ovariectomy and simultaneously analyzed to what extent these changes could be reversed by 17β-estradiol.

## 2. Materials and Methods

### 2.1. Animals

The HHTg rats were provided by the Institute for Clinical and Experimental Medicine (Prague, Czech Republic). All experiments were performed in agreement with the Animal Protection Law of the Czech Republic (359/2012) and the Directive 2010/63/EU of the European Parliament and of the Council, and were approved by the Ethics Committee of the Institute for Clinical and Experimental Medicine, Prague (Protocol Number: 6/2020).

The rats were housed in a 12 h light/12 h dark cycle room at a temperature of 22–25 °C and allowed free access to a standard chow diet (Altromin, Maintenance diet for rats and mice, Lage, Germany) and drinking water. The HHTg female rats were randomly divided into three experimental groups (*n* = 6 in each group) at the beginning of the study. Two-month-old HHTg females underwent bilateral ovariectomy (OVX) under anesthesia (intramuscular injection of ketamine/xylazin mixture (70/10 mg/kg body weight)). During the procedure, the females were saturated with oxygen followed by subcutaneous analgesia (meloxicam 1 mg/kg). Control rats (SHAM) underwent the entire surgery without the removal of their ovaries. After surgery, the health status of the rats was monitored daily. Two weeks after surgery, half of the ovariectomized females were substituted with 17-β estradiol (Sigma-Aldrich, St. Louis, MO, USA) subcutaneously at a dose 12.5 μg/kg body weight per day for twelve weeks (OVX+E2).

At the end of the study, the females were decapitated under anesthesia (zoletil 5 mg/kg body weight) in a postprandial state, and blood serum and tissue samples were collected and stored at −80 °C for subsequent analysis. During the experiment, body weight and food intake were measured weekly.

### 2.2. Biochemical Analysis

The serum concentrations of glucose, triglycerides (TG), nonesterified fatty acids (NEFA), and total cholesterol were measured using commercially available kits (Erba Lachema, Brno, Czech Republic). Serum insulin and monocyte chemoattractant protein-1 (MCP-1) were analyzed using a Rat Insulin ELISA kit (Mercodia AB, Uppsala, Sweden) and a Rat MCP-1 Instant ELISA kit (eBioscience, Vienna, Austria), respectively. High-sensitivity C-reactive protein (hsCRP), leptin, ghrelin, and high-molecular-weight (HMW) adiponectin were also measured using rat ELISA kits (BioVendor, Brno, Czech Republic and MyBioSource, San Diego, CA, USA). 17β-estradiol was determined by an Ultra-sensitive Estradiol RIA kit (Immunotech, Prague, Czech Republic).

For the oral glucose tolerance test (OGTT), blood glucose was determined after a glucose load (3 g of glucose/kg body weight) was administered intragastrically after overnight fasting. Glucose concentrations were determined by analyzing the blood samples collected from the tail vein before the glucose load (0 min) and 30, 60, 120, and 180 min after glucose loading. The area under the glycemic curve (AUC) was calculated over a 180 min period.

The adiposity index is expressed as the sum of the weights of the perimetrial and perirenal adipose tissues divided by body weight.

For TG determination in the liver, kidney, and muscles (*musculus gastrocnemius*), samples were powdered under liquid N_2_ and extracted in chloroform/methanol. The mixture was centrifuged, and the organic phase was removed and evaporated under N_2_. The resulting pellet was dissolved in isopropyl alcohol, and the TG concentration was measured by an enzymatic assay (Erba-Lachema, Brno, Czech Republic).

### 2.3. Tissue Insulin Sensitivity

Peripheral tissue insulin sensitivity was measured ex vivo according to the insulin-stimulated incorporation of glucose into glycogen in the diaphragm or lipids in the perimetrial adipose tissue. The diaphragm or distal parts of the perimetrial adipose tissue were dissected and immediately incubated for 2 h in Krebs-Ringer bicarbonate buffer (pH 7.4) containing 0.1 μCi/mL of ^14^C-U glucose, 5.5 mM unlabeled glucose, and 2.5 mg/mL of bovine serum albumin (Sigma-Aldrich, St. Louis, MO, USA) without or with 250 μU/mL of insulin at 37 °C. The extraction of glycogen and lipids was followed by a determination of the basal and insulin-stimulated incorporation of glucose into glycogen or lipids, as previously described [23]. In the case of glycogenesis determination, the diaphragm was boiled in 30% KOH with 1% glycogen, then 96% ethanol was added and the mixture was centrifuged. The sediment was dissolved in H_2_O and the radioactivity was measured by scintillation counting in Rotiszint solution (Roth, Karlsruhe, Germany). The glycogenesis is expressed in nmols of ^14^C-glycogen per gram of diaphragm tissue per 2 h (nmol ^14^C-gl/g/2 h). In the case of lipogenesis, pieces of perimetrial adipose tissue were taken out from the buffer, rinsed in saline, and placed into chloroform:methanol (2:1), where lipids were extracted at 4 °C overnight. Then, KH_2_PO_4_ was added before evaporating and reconstituting the aliquots in scintillation liquid. Radioactivity was measured by scintillation counting in Rotiszint. The lipogenesis is expressed in nmols of ^14^C-TG per gram of adipose tissue per 2 h (nmol ^14^C-TG/g/2 h).

Basal and adrenaline-stimulated lipolysis in the perimetrial adipose tissue was measured ex vivo based on the release of NEFA into the incubation medium. Briefly, distal parts of the perimetrial adipose tissue were incubated for 2 h in Krebs-Ringer phosphate buffer containing 3% bovine serum albumin at 37 °C, pH 7.4, with or without adrenaline (0.25 μg/mL). After incubation, the concentrations of NEFA in the medium were determined (Erba Lachema, Brno, Czech Republic).

The content of proteins in perimetrial adipose tissue was analyzed according to Lowry et al. [24].

### 2.4. Transcriptomic Analysis

The total mRNA was isolated from the perimetrial adipose tissue using an RNeasy Plus Mini Kit (Qiagen, Valencia, CA, USA). The quality and integrity of the total RNA were evaluated on an Agilent 2100 Bioanalyzer system (Agilent, Palo Alto, CA, USA), and only samples with an RNA Integrity Number (RIN) > 8.0 were utilized in further steps (SHAM: *n* = 5; OVX: *n* = 6; OVX+E2: *n* = 6). Microarray experiments were performed using the Affymetrix^®^ Rat Gene 2.1 ST Array Strip (Affymetrix, Santa Clara, CA, USA). The hybridization procedure was performed using the Affymetrix GeneAtlas^®^ system according to the manufacturer’s instructions. The quality control of the chips was performed using Affymetrix Expression Console software v.1.0.1. (Affymetrix, Santa Clara, CA, USA), and for subsequent data analysis, Partek Genomics Suite 7 (Partek, St. Louis, MO, USA) was used. After applying quality filters and data normalization by the Robust Multichip Average (RMA) algorithm, the obtained set of differentially expressed probe sets was filtered by the false discovery rate (FDR) method that is implemented in Partek Genomics Suite 7 (Partek, St. Louis, MO, USA). Only probe sets with an FDR < 0.1 and, at the same time, showing a >1.2 fold or <−1.2 fold difference in expression between the control and experimental groups, were subjected to further analyses. Transcriptomic data underwent a standardized sequence of analyses, including hierarchical clustering, principal component analysis, gene ontology, gene set enrichment, upstream regulator analysis, mechanistic networks, causal network analysis, and downstream effects analysis, utilizing the Partek Pathway, Ingenuity Pathway Analysis (Qiagen), and enrichment analysis tool Enrichr [25]. The generated and analyzed microarray data from this study can be accessed in the EMBL-EBI Biostudies repository (https://www.ebi.ac.uk/biostudies/ accessed on 14 May 2024) under accession number E-MTAB-13224.

### 2.5. Quantitative Real-Time PCR

For validation of the gene expression data obtained by microarray, quantitative real-time PCR (qPCR) was performed. Reverse transcription and qPCR analyses were carried out using the TaqMan RNA-to-CT 1-Step Kit, TaqMan Gene Expression Assay (Applied Biosystems, Waltham, MA, USA) and ViiA^TM^ 7 Real-Time PCR System (Applied Biosystems). The following TaqMan probes were used: stearoyl-CoA-desaturase-1 (*Scd-1*): Rn00594894_g1, cytokine inducible SH2 containing protein (*Cish*): Rn01446958_g1, serpin family E member 1 (*Serpine 1*): Rn01481341_m1, and crystallin alpha B (*Cryab*): Rn01421541_m1. The relative expressions were determined after normalization against hypoxanthine phosphoribosyltransferase 1 (*Hprt1*): Rn01527840_m1 as an internal reference and calculated using the 2^−ΔΔCt^ method, with results run in triplicate (Supplementary Figure S1).

### 2.6. Statistical Analysis

The data are expressed as mean ± standard error of the mean (SEM). The morphometric and biochemical data obtained in this study were analyzed using TIBCO Statistica ^TM^ 14.00 software (TIBCO Software Inc., Palo Alto, CA, USA). One-way ANOVA was used to analyze the effect of ovariectomy and 17β-estradiol treatment, and for detailed comparisons, LSD-Fisher’s post hoc test was applied. Statistical significance was defined as *p* < 0.05.

## 3. Results

### 3.1. Effect of Ovariectomy and 17β-Estradiol Substitution on Body Weight, Food Intake, and Glucose Tolerance

**Figure 1:**
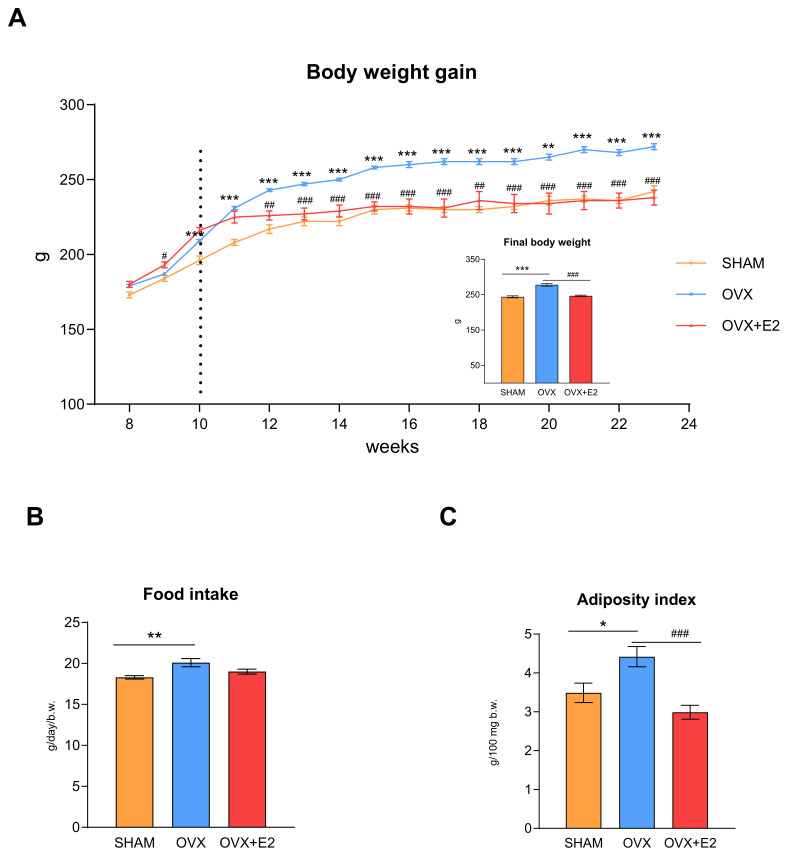
Body weight gain during the experiment and final body weight (**A**), food intake (**B**), and visceral adiposity index (**C**) in sham-operated (SHAM), ovariectomized (OVX), and ovariectomized HHTg rats treated with 17β-estradiol (OVX+E2). Data are expressed as mean ± SEM; *n* = 6 for each group. * *p* < 0.05, ** *p* < 0.01, and *** *p* < 0.001 denote significant differences between SHAM vs. OVX and OVX+E2 groups, ^#^ *p* < 0.05, ^##^ *p* < 0.01, and ^###^ *p* < 0.001 denote significant differences between OVX vs. OVX+E2 groups. The dotted line in Panel A indicates the beginning of 17β-estradiol substitution.

The initial body weights of the rats at 8 weeks of age did not differ among the experimental groups (Figure 1A). From the age of 10 weeks (2 weeks after ovariectomy), the OVX females showed an increased body weight gain compared to the SHAM group, which persisted throughout the study (i.e., until 23 weeks of rat age). This increased body weight gain in the OVX females was associated with a 10% increase in their food intake (Figure 1B). At 12 weeks of age (2 weeks after initiation of 17β-estradiol treatment), the body weight gain in OVX+E2 females was significantly lower compared to that in the OVX females (Figure 1A). This difference in weight gain between the OVX and OVX+E2 groups was maintained until the end of the experiment, although food intake did not differ between these experimental groups.

As shown in Figure 1C, the adiposity index, a measure of visceral obesity, was higher in the ovariectomized rats compared to the other groups. 17β-estradiol substitution markedly reduced the adiposity index, as well as the final body weight, in the OVX+E2 rats.

**Figure 2:**
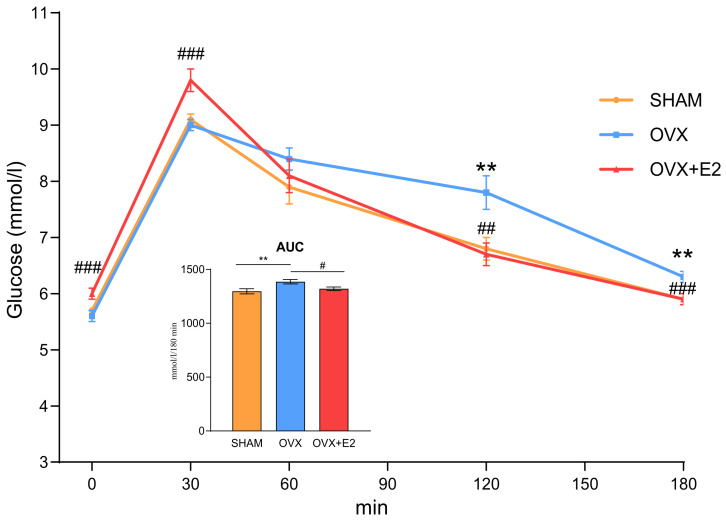
Oral glucose tolerance test in sham-operated (SHAM), ovariectomized (OVX), and ovariectomized HHTg rats treated with 17β-estradiol (OVX+E2). Data are expressed as mean ± SEM; *n* = 6 for each group. ** *p* < 0.01 denotes significant difference between SHAM vs. OVX groups, ^#^ *p* < 0.05, ^##^ *p* < 0.01, and ^###^ *p* < 0.001 denote significant difference between OVX vs. OVX+E2 groups. AUC—area under the curve during the oral glucose tolerance test.

Ovariectomy in the HHTg rats was accompanied by an impaired glucose tolerance, as evidenced by OGTT (Figure 2). 17β-estradiol substitution improved the glucose tolerance in the ovariectomized females, with significantly lower glycemia in the OVX+E2 compared to the OVX group in all but one (60th minute) analyzed time points of the OGTT.

### 3.2. Effect of Ovariectomy and 17β-Estradiol Substitution on Serum Metabolic Parameters, Inflammatory Markers, and Ectopic Lipid Accumulation

As expected, ovariectomy resulted in a decreased serum level of 17β-estradiol, whereas 17β-estradiol replacement in the OVX rats markedly increased the serum levels of this sex hormone (Table 1).

The ovariectomized HHTg females exhibited no changes in their non-fasting serum glucose, insulin, cholesterol, NEFA, HMW adiponectin, ghrelin, pro-inflammatory chemokine MCP-1, and hsCRP compared to the sham-operated controls. The administration of 17β-estradiol to the ovariectomized HHTg rats had no additional impact on these serum parameters, except for TG and cholesterol, the levels of which increased after 17β-estradiol treatment. After ovariectomy, serum leptin levels were significantly increased and remained unchanged in the OVX+E2 group (Table 1).

**Figure 3:**
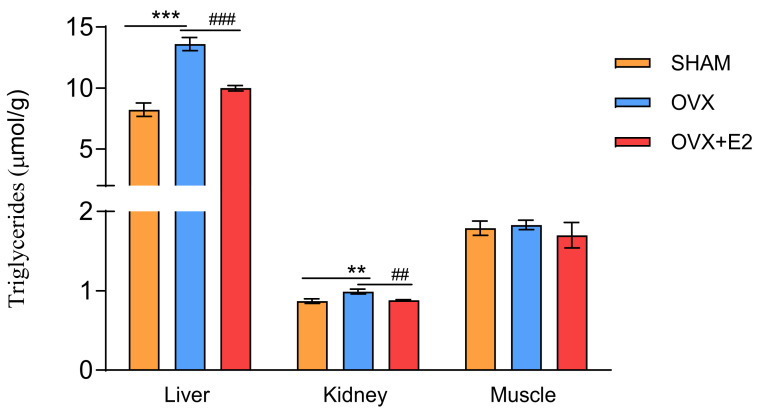
Ectopic triglycerides (TG) accumulation in liver, kidney, and skeletal muscle in sham-operated (SHAM), ovariectomized (OVX), and ovariectomized HHTg rats treated with 17β-estradiol (OVX+E2). Data are expressed as mean ± SEM; *n* = 6 for each group. ** *p* < 0.01 and *** *p* < 0.001 denote significant differences between SHAM vs. OVX groups, ^##^ *p* < 0.01 and ^###^ *p* < 0.001 denote significant differences between OVX vs. OVX+E2 groups.

Ovariectomy in the HHTg rats resulted in significant ectopic deposition of TG in the liver and kidneys (Figure 3) and a concomitant decrease in serum TG (Table 1). 17β-estradiol substitution reduced TG accumulation in the liver by 21% and in the kidneys by 23%, while simultaneously raising serum TG levels. On the other hand, neither ovariectomy nor the 17β-estradiol administration to the ovariectomized HHTg females affected TG accumulation in the muscle (Figure 3).

### 3.3. Effect of Ovariectomy and 17β-Estradiol Substitution on Insulin Sensitivity of Peripheral Tissues

**Figure 4:**
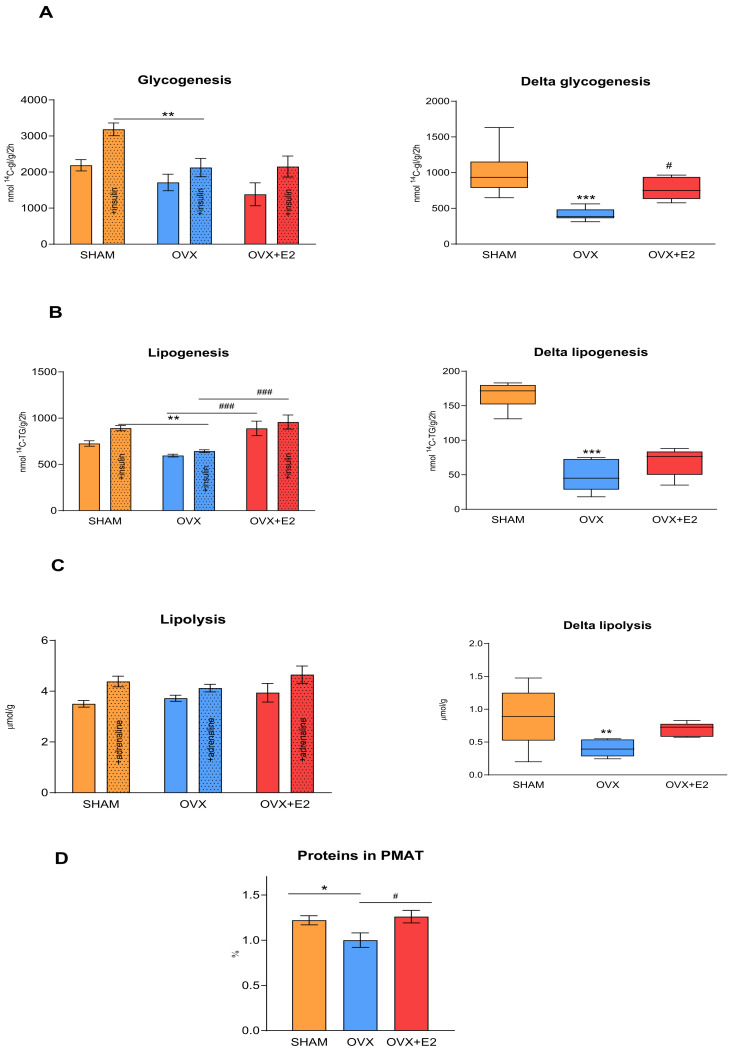
Muscle (**A**) and perimetrial adipose tissue (**B**) sensitivity to insulin action, basal and adrenaline-stimulated lipolysis from perimetrial adipose tissue (**C**), and protein content in perimetrial adipose tissue (**D**) in sham-operated (SHAM), ovariectomized (OVX), and ovariectomized HHTg rats treated with 17β-estradiol (OVX+E2). The graphs in the right-hand column show the absolute differences (Delta) between basal and stimulated conditions (**A**–**C**). Data are expressed as mean ± SEM; *n* = 6 for each group. * *p* < 0.05, ** *p* < 0.01, and *** *p* < 0.001 denote significant differences between SHAM vs. OVX groups, ^#^ *p* < 0.05 and ^###^ *p* < 0.001 denote significant differences between OVX vs. OVX+E2 groups. PMAT—perimetrial adipose tissue.

Figure 4 shows the basal and insulin-stimulated glucose incorporation into the muscle glycogen and lipids of perimetrial adipose tissue. Ovariectomy led to a decrease in insulin-stimulated glycogenesis (Figure 4A). Delta glycogenesis, which represents the difference between basal and insulin-stimulated glycogenesis, was significantly lower in the OVX compared to SHAM group. 17β-estradiol administration to the OVX females partially restored delta glycogenesis, indicating an increased muscle insulin sensitivity in the OVX+E2 rats in comparison to the OVX rats.

As shown in Figure 4B, the hypertriglyceridemic females after ovariectomy displayed a reduced insulin-stimulated sensitivity in their perimetrial adipose tissue, which is representative of visceral adipose tissue. 17β-estradiol substitution in the OVX rats improved the basal as well as stimulated insulin sensitivity of the perimetrial adipose tissue, but did not affect delta lipogenesis. On the other hand, delta lipogenesis in the OVX females was significantly reduced compared to in the SHAM rats, suggesting that, in the OVX rats, perimetrial adipose tissue was almost resistant to insulin action.

Both basal and adrenaline-stimulated lipolysis in the perimetrial adipose tissue did not differ among the experimental groups (Figure 4C). Delta lipolysis in the OVX rats was reduced compared to the SHAM rats and was not restored by 17β-estradiol administration, showing perimetrial adipose tissue to be resistant to adrenaline stimulation in ovariectomized rats. A decreased protein content in the perimetrial adipose tissue in the OVX rats may be related to the decreased metabolic activity of the perimetrial adipose tissue and the early development of adipose tissue insulin resistance (Figure 4D). 17β-estradiol substitution in the OVX+E2 rats reversed this effect, increasing the protein content in the perimetrial adipose tissue compared to that in the OVX rats.

### 3.4. Effect of Ovariectomy and 17β-Estradiol Substitution on Gene Expression Profiles of Perimetrial Adipose Tissue

**Figure 5:**
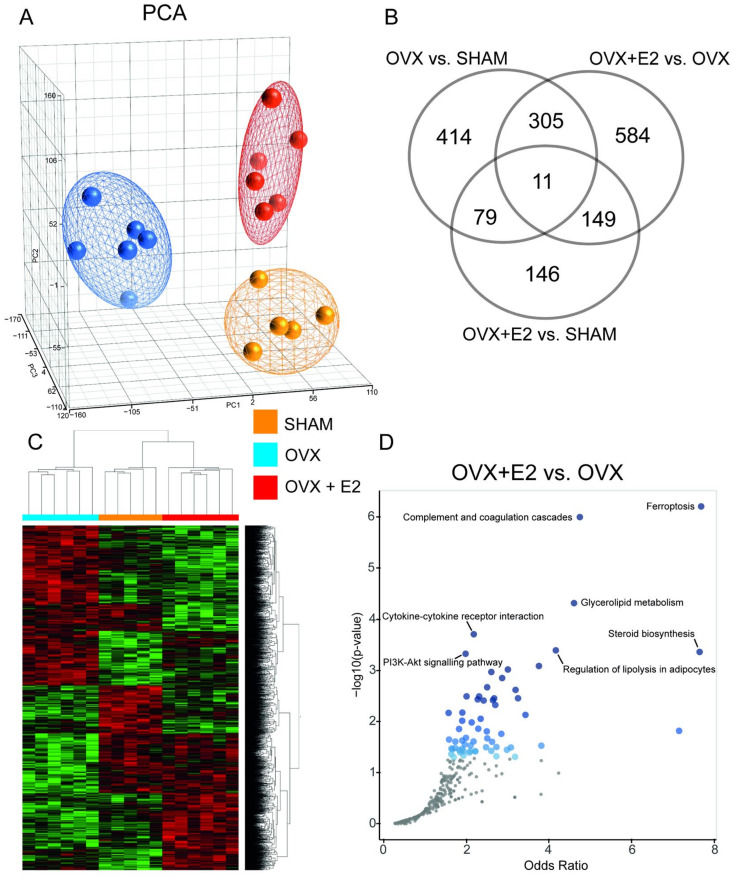
Overview of transcriptome of perimetrial adipose tissue of sham-operated (SHAM), ovariectomized (OVX), and ovariectomized HHTg rats treated with 17β-estradiol (OVX+E2). (**A**) Principal component analysis (PCA) of transcriptome in all three groups; SHAM shown in orange, OVX shown in blue, and OVX+E2 shown in red. (**B**) Venn diagram of numbers of significantly differentially expressed genes (DEGs) among the individual pairwise comparisons. The lists of the differentially expressed genes pertaining to the particular overlaps are shown in Supplementary Table S4. (**C**) Hierarchical clustering of DEGS in all samples. (**D**) Volcano plot of terms from the KEGG Pathway database. Each point represents a single term, plotted by the corresponding odds ratio (x-position) and −log10(*p*-value) (y-position) from the enrichment results of the DEGs set of OVX+E2 vs. OVX comparison. The larger and darker-colored the point, the more significantly enriched the input gene set is for the term. The name labels are provided only for the pathways passing the adjusted significance level (Benjamini–Hochberg; q < 0.05).

A transcriptomic analysis of the perimetrial adipose tissue revealed distinct expression profiles among the three groups, with the OVX rats showing the most divergence from the other two groups, as evidenced by the results of the principal component and hierarchical clustering analyses (Figure 5). There were 809 differentially expressed genes (DEGs) in the ovariectomized rats compared to the SHAM rats (350 up- and 459 down-regulated in OVX vs. SHAM, respectively; Supplementary Table S1), mostly pertaining to the regulation of the lipid and glucose metabolism, cell cycle, and oxidative stress. We identified a significant overrepresentation in four pathways from the Kyoto Encyclopedia of Genes and Genomes (KEGG) based on the OVX vs. SHAM DEGs, including the FoxO (Benjamini–Hochberg (B-H) adjusted q = 0.025) and PI3K-Akt signaling (B-H q = 0.042) pathways (Supplementary Figure S2). 17β-estradiol substitution in the ovariectomized HHTg rats affected 1049 transcripts (540 up- and 509 down-regulated in OVX+E2 vs. OVX, respectively; Supplementary Table S2) with significant overrepresentation in seven KEGG pathways, including ferroptosis (B-H q = 0.0002), glycerolipid metabolism (B-H q = 0.007), steroid biosynthesis (B-H q = 0.025), regulation of lipolysis in adipocytes (B-H q = 0.026), and the PI3K-Akt signaling pathway (B-H q = 0.039), as shown in Figure 5. The expression of the selected genes was validated by quantitative real-time PCR and, in all cases, the differences in their relative expressions were confirmed (Supplementary Figure S1). While there were 385 DEGs between the OVX+E2 and SHAM groups (186 up- and 199 down-regulated in OVX+E2; Supplementary Table S3), there were no significantly enriched pathways based on this dataset (Supplementary Figure S3). Almost all DEGs after ovariectomy in the perimetrial adipose tissue that were also affected by 17β-estradiol administration showed an opposite direction of expression change between the two conditions (Supplementary Figure S4). For example, Scd-1, a key enzyme in monounsaturated fatty acid synthesis, was one of the most downregulated genes after ovariectomy (−5.97 fold in OVX vs. SHAM, *p* = 3.78 × 10^−6^). 17β-estradiol administration to the OVX females significantly upregulated its expression (3.09 fold in OVX+E2 vs. OVX, *p* = 2.57 × 10^−4^).

## 4. Discussion

Menopause is associated with an increased risk of metabolic disease, including type 2 diabetes and cardiovascular diseases, which are among the leading causes of death in postmenopausal women worldwide [26]. Menopause induced by surgical ovariectomy provides an appropriate tool for studying menopausal disorders in animal models. In our study, on the background of insulin resistance in HHTg rats, we investigated the effects of menopause and 17β-estradiol substitution on the metabolic parameters, insulin sensitivity, and transcriptome of perimetrial adipose tissue. This study showed that an ovariectomy-induced worsening of the metabolic disorders accompanying metabolic syndrome in female rats could be partially reversed by 17β-estradiol replacement.

Ovariectomy-induced menopause in the HHTg females was accompanied by increased body weight gain and adiposity index, the marker of visceral fat. A loss of ovarian hormones increases food intake and body weight in humans and rodents and contributes to the increase in adipose tissue mass [9,12,16]. The elevated circulating leptin levels found in our study may be related to the increased food intake and associated weight gain after ovariectomy. Leptin regulates food intake and energy expenditure, thereby maintaining energy homeostasis. Its levels tend to be elevated in obesity and reflect the volume of fat mass [9]. On the other hand, ovariectomized rats treated with 17β-estradiol had a constantly lower body weight gain compared to the OVX females. A reduced final body weight in the OVX+E2 rats corresponded with a significantly reduced adiposity index, although their food intake was unchanged. Kawakami et al. demonstrated an inhibitory effect of estrogen against abdominal obesity in ovariectomized rats [10]. Similarly, estrogen replacement in postmenopausal women prevented obesity [27]. Estrogen could directly inhibit adipose tissue deposition by decreasing lipogenesis [28]. These findings indicate that estrogens play an important role in the regulation of adipose tissue development and deposition.

While ovariectomy did not impact glycemia and insulinemia, it impaired glucose tolerance in the HHTg rats, as assessed by the OGTT. Conversely, the administration of 17β-estradiol to the ovariectomized rats resulted in an improvement in glucose tolerance. Estrogens are regulators of energy balance and glucose metabolism, play a role in insulin-induced glucose transport and hepatic glucose output, and their deficiency strongly contributes to impaired glucose metabolism [28,29,30] Moreover, each year after menopause increases the risk of impaired glucose tolerance by 6% [29]. In many human and animal studies, estradiol administration has improved the impaired glucose tolerance occurring in postmenopausal period [31,32,33]. Hormone replacement therapy improves insulin secretion, glucose effectiveness, and insulin sensitivity [32].

In women, menopause is associated with proatherogenic changes in the lipid profile, which includes a high total cholesterol, LDL cholesterol, TG, and low HDL cholesterol [34]. The severity of dyslipidemia stems from its role as a risk factor for CVD. However, the effect of ovariectomy-induced menopause on serum TG is inconsistent in animal studies. Increased total cholesterol without changes in TG levels, as well as no changes in either parameters or reduced serum TG levels, have been reported [17,35,36]. In our study, we observed a decrease in serum TG and no changes in circulating cholesterol levels after ovariectomy. In addition, ovariectomy in the HHTg rats was accompanied by significant ectopic TG accumulation in the liver and kidney, and 17β-estradiol substitution markedly reduced TG deposition while simultaneously increasing serum TG levels. The hepatic TG accumulation observed in the OVX females and concomitantly reduced circulating TG may indicate that TG was deposited in the liver. In a number of trials and prospective studies, hormone replacement therapy increased plasma TG levels, despite improvement in a number of risk factors for CVD [37,38,39,40,41]. Several studies have demonstrated that this TG level increase is due to increased VLDL production during estrogen treatment [41,42]. A similar effect was observed after 17β-estradiol treatment in ovariectomized rats, which is consistent with our present and previous study [35,43]. In the liver, increased TG accumulation leads to hepatic steatosis and strongly contributes to hepatic insulin resistance. Various mechanisms are involved in lipid accumulation in the liver, including an increased hepatic uptake of circulating fatty acids and de novo fatty acids synthesis and decreased hepatic beta-oxidation and lipid exports [44]. On a molecular level, hepatic lipid deposition is facilitated by transcription factor peroxisome proliferator-activated receptor-γ (PPARγ), which is essential for adipogenesis and lipid storage. PPARγ elevated levels have been observed in murine models with hepatic steatosis and its overexpression in hepatocytes turns on a program that increases the expression of lipogenic genes [45,46]. Hepatic steatosis caused by the absence of ovarian hormones could be at least partially reversible with estrogen replacement. The mechanisms by which estrogen protects against hepatic steatosis include suppression in liver lipogenesis (by maintaining acetyl-CoA carboxylase (ACC) phosphorylation) and the promotion of fatty acid oxidation in the liver [47,48].

In addition to participating in the regulation of lipid metabolism, estrogens also modulate insulin sensitivity. A deficiency of estrogens is associated with the dysregulation of metabolic homeostasis and insulin resistance and contributes to the development of type 2 diabetes and obesity in both human and animal models [48,49,50]. However, the tissue-specific effects of estrogens on metabolic changes and their underlying mechanisms are not fully understood. We analyzed tissue insulin sensitivity according to the incorporation of radiolabeled glucose into the muscle glycogen or lipids of the perimetrial adipose tissue. In the OVX females, insulin-stimulated glycogenesis was reduced, corresponding to a markedly reduced delta glycogenesis, although muscle TG was unchanged. This suggests that the reduction in muscle insulin sensitivity in the HHTg females after menopause was not caused by ectopic TG accumulation. Since skeletal muscle plays a key role in insulin-stimulated glucose uptake, it contributes significantly to the development of insulin resistance. 17β-estradiol administration to ovariectomized rats partially restored muscle insulin sensitivity, as indicated by increased delta glycogenesis. Perimetrial adipose tissue, a representative of visceral fat, is fundamentally involved in the changes occurring after menopause. We observed a reduction in protein content in the perimetrial adipose tissue of the OVX females, suggesting a shift from small, metabolically active adipocytes to larger, less active ones. This was accompanied by markedly decreased lipogenesis and lipolysis, indicating insulin resistance and significantly reduced metabolic activity in the perimetrial adipose tissue. Conversely, 17β-estradiol increased protein content and improved basal and insulin-stimulated lipogenesis. The HHTg strain of rats is characterized by chronically elevated levels of TG and NEFA compared to the control Wistar rats [51]. NEFA contributes to lipotoxicity in tissues by generating lipotoxic intermediates and promoting insulin resistance. However, in our study, we did not observe changes in circulating NEFA either due to ovariectomy or to 17β-estradiol substitution.

Although the mechanisms underlying reduced insulin sensitivity are multifactorial and not fully understood, it is widely accepted that insulin resistance is associated with excess visceral adipose tissue and inflammation. Adipokines, which are secreted by adipose tissue, modulate inflammation, adiposity, and glucose and lipid metabolism [52]. In our study, despite observing significant changes in perimetrial adipose tissue insulin sensitivity, there were no effects on the circulating levels of pro-inflammatory MCP-1 or hsCRP, and the levels of HMW adiponectin remained unchanged. Our findings are consistent with other studies that have reported no changes in adiponectin levels in ovariectomized and estradiol-treated rats [36,53]. Adiponectin has been shown to affect insulin sensitivity through the modulation of insulin signaling and the molecules involved in glucose and lipid metabolism, and its reduced levels have been connected with obesity and insulin resistance [54,55]. In addition, HMW adiponectin has anti-inflammatory and anti-atherogenic properties [7]. Our results suggest that the decreased insulin sensitivity was not associated with a pro-inflammatory state in the perimetrial adipose tissue. This is supported by the absence of gene expression changes in essential pro-inflammatory factors. This finding contrasts with several rodent studies that have shown the development of adipose tissue inflammation following ovariectomy [45,56]. Impaired insulin sensitivity of perimetrial adipose tissue may be related to markedly increased levels of leptin and associated leptin resistance. Most studies of ovariectomized rodents have found elevated serum leptin levels consistent with ours [7,9,52].

Ovariectomy-induced menopause had a large impact on the perimetrial adipose tissue, therefore, we studied ovariectomy-induced shifts in the global gene expression and the effect of 17β-estradiol substitution. There was an evident, significant shift in gene expression due to ovariectomy, which was partially reversed along the same principal component by 17β-estradiol substitution. Still, there was a clear distinction between the OVX+E2 and SHAM groups, suggesting that not all changes caused by ovariectomy can be restored by 17β-estradiol substitution. One of the most overrepresented pathways was ferroptosis, downregulated by 17β-estradiol administration. This supports the recent observation that estrogen suppresses endothelial cell ferroptosis via inhibiting lipid peroxidation and iron accumulation, decreasing reactive oxygen species production, and improving glutathione (GSH) levels and mitochondrial function [57]. Lipids and lipid metabolism can shape the cell’s sensitivity to ferroptosis, with the particular importance of a balancing system comprising two long-chain fatty acyl-CoA synthetase (Acsl) enzymes, Acsl3 and Acsl4, preferentially incorporating monounsaturated and polyunsaturated fatty acids into phospholipids, respectively [58]. In the perimetrial adipose tissue of the OVX+E2 rats, we observed the significant upregulation of *Acsl3* and no change in *Acsl4* expression compared to the OVX group, corroborating its protective effect against lipoperoxidation and ferroptosis [58]. The changes in the expression of the glycerolipid metabolism genes corresponded to the results of the metabolic profile and diminishing fat depots with the downregulation of genes involved in several steps of lipogenesis (including e.g., diacylglycerol O-acyltransferase 2, lipin 2, or 1-acylglycerol-3-phosphate O-acyltransferase 4) and a concomitant shift in the expression profile corresponding to limited lipolysis in adipocytes.

Some of the genes upregulated in the ovariectomized rats (and, in turn, downregulated by 17β-estradiol administration) were two adipokines, crystallin alpha B (*Cryab*) and *Serpine1*. *Cryab* expression is strongly induced during adipogenesis and its expression in the visceral adipose tissue of obese subjects has been shown to be significantly higher compared with lean controls [59], possibly explaining its changes in the current study, along with the changes in the adipose tissue mass. Similarly, the expression of *Serpine1* was increased in the adipocytes of obese mice when assessed by single-nucleus RNA-seq [60]. The deterioration of glucose tolerance in the OVX rats was accompanied by significant downregulation of the FoxO and PI3K-Akt signaling pathways. The genes most downregulated in the ovariectomized rats that had their expression, in turn, significantly reversed by 17β-estradiol were cytokine-inducible SH2-domain-containing protein (*Cish*) and stearoyl-CoA desaturase-1 (*Scd-1*). The short-term administration of 17β-estradiol to the ovariectomized mice was not reported to change the *Scd-1* expression in murine perimetrial adipose tissue [61,62]. Our observation of a decrease in *Scd-1* expression after ovariectomy and its subsequent increase after 17β-estradiol supplementation is in contrast with the evidence for the ability of estrogen to repress the Scd-1 activity and expression. On the other hand, it was shown that estrogen induced Scd-1 expression and activity in breast carcinoma cells [63]. Our seemingly paradoxical result may be explained by the alignment of the observed decreased lipogenesis and activity of the perimetrial adipose tissue with a concomitant decrease in *Scd-1* expression in the OVX rats. Also, the increased concentration of leptin could repress the *Scd-1* expression [64]. As Sun et al. recently reviewed, Scd-1 is a pivotal metabolic homeostasis regulator, often displaying dual roles in adiposity and insulin resistance [65]. Previous observations regarding the role of Cish in metabolism have been controversial. *Cish* knockout mice showed an improved insulin sensitivity and were resistant to diet-induced obesity [66]. On the other hand, adenovirus-mediated hepatic *Cish* knockdown and liver-specific *Cish* knockout impaired the glucose tolerance and increased gluconeogenesis in WT mice [67].

While changes in the mRNA levels of lipid metabolism enzymes offer valuable insights, it should be acknowledged that they do not necessarily reflect enzymatic activities, thus warranting further investigation to determine how these changes translate into functional enzymatic activity following ovariectomy and estradiol treatment.

## 5. Conclusions

In summary, our study demonstrated that ovariectomy-induced menopause in HHTg rats impaired the glucose tolerance, potentiated ectopic lipid deposition, and worsened the insulin resistance of peripheral tissues. Changes in the perimetrial adipose tissue transcriptome, particularly in pathways relevant to lipid metabolism and oxidative stress, may contribute to metabolic abnormalities accompanying metabolic syndrome in postmenopausal HHTg females. 17β-estradiol substitution may partially reverse some of the metabolic disorders associated with ovariectomy. This study provides valuable insights into the mechanisms underlying the increased risk of metabolic disorders associated with menopause.

## Figures and Tables

**Table 1:** Effect of ovariectomy and 17-β estradiol substitution on basal metabolic parameters in serum of hereditary hypertriglyceridemic rats.

	SHAM	OVX	OVX+E2	P_ANOVA_
**Non-fasting glucose (mmol/L)**	7.867 ± 0.319	8.367 ± 0.154	8.067 ± 0.287	n.s.
**Insulin (nmol/L)**	0.217 ± 0.025	0.195 ± 0.014	0.167 ± 0.010	n.s.
**Triglycerides (mmol/L)**	3.557 ± 0.300	1.720 ± 0.105 ***	3.298 ± 0.137 ^###^	<0.001
**Cholesterol (mmol/L)**	1.665 ± 0.104	1.849 ± 0.045	2.177 ± 0.064 ^##^	<0.001
**NEFA (mmol/L)**	0.611 ± 0.057	0.648 ± 0.032	0.654 ± 0.028	n.s.
**17β-estradiol (pg/mL)**	28.951 ± 3.913	15.062 ± 1.156	283.598 ± 32.489 ^###^	<0.001
**Leptin (ng/mL)**	5.311 ± 0.229	7.322 ± 0.322 ***	7.526 ± 0.360	<0.001
**Ghrelin (pg/mL)**	248.167 ± 63.016	98.833 ± 32.149	566.000 ± 281.889	n.s.
**HMW adiponectin (μg/mL)**	2.702 ± 0.130	3.336 ± 0.387	3.144 ± 0.226	n.s.
**MCP-1 (ng/mL)**	5.297 ± 0.473	6.135 ± 0.270	7.383 ± 1.666	n.s.
**hsCRP (mg/mL)**	1.194 ± 0.034	1.278 ± 0.217	1.571 ± 0.098	n.s.

Data are expressed as mean ± SEM; *n* = 6 for each group. P_ANOVA_ (One-way ANOVA) denotes significant difference one of the three compared groups, for detailed analysis was used Fisher LSD post-hoc test, *** *p* < 0.001 denotes significant difference between SHAM vs. OVX groups, ## *p* < 0.01 and ### *p* < 0.001 denote significant differences between OVX vs. OVX+E2 groups. NEFA– nonesterified fatty acids, HMW adiponectin—high-molecular-weight adiponectin, MCP-1—monocyte chemoattractant protein-1, hsCRP—high-sensitivity C-reactive protein, n.s.—not significant difference.

## Data Availability

All data generated during this study are included in the article and its additional files. The generated and analyzed microarray data from this study can be accessed in the EMBL-EBI Biostudies repository (https://www.ebi.ac.uk/biostudies/ accessed on 14 May 2024) under accession number E-MTAB-13224.

## Supplementary Materials

The following supporting information can be downloaded at: https://www.mdpi.com/article/10.3390/antiox13060627/s1, Figure S1: Quantitative real-time PCR (qPCR) validation of selected significantly upregulated and downregulated genes; Figure S2: Volcano plot of terms from the KEGG Pathway database (OVX vs. SHAM); Figure S3: Volcano plot of terms from the KEGG Pathway database (OVX+E2 vs. SHAM); Figure S4: Detailed comparison of 316 differentially expressed genes overlapping between datasets comparing the expression in ovariectomized rats vs. sham-operated HHTg female rats and in OVX substituted with 17β-estradiol vs. OVX; Table S1: Summary of the significantly differentially expressed genes (FDR < 0.05, fold-change > 1.2 or <−1.2) in perimetrial adipose tissue of ovariectomized (OVX) and sham-operated (SHAM) female HHTg rats, based on the transcriptomic profiling using the Affymetrix Rat Gene 2.1 ST Array Strip; Table S2: Summary of the significantly differentially expressed genes (FDR < 0.05, fold-change > 1.2 or <−1.2) in perimetrial adipose tissue of ovariectomized female HHTg rats receiving 17β-estradiol (OVX+E2) and ovariectomized female HHTg rats (OVX), based on the transcriptomic profiling using the Affymetrix Rat Gene 2.1 ST Array Strip; Table S3: Summary of the significantly differentially expressed genes (FDR < 0.05, fold-change > 1.2 or <−1.2) in perimetrial adipose tissue of ovariectomized female HHTg rats receiving 17β-estradiol (OVX+E2) and sham-operated female HHTg rats (SHAM), based on the transcriptomic profiling using the Affymetrix Rat Gene 2.1 ST Array Strip. Table S4: Summary of overlapping and unique sets of the significantly differentially expressed genes (FDR < 0.05, fold-change > 1.2 or <−1.2) in perimetrial adipose tissue of ovariectomized female HHTg rats (OVX), ovariectomized female HHTg rats receiving 17β-estradiol (OVX+E2) and sham-operated female HHTg rats (SHAM), based on the transcriptomic profiling using the Affymetrix Rat Gene 2.1 ST Array Strip.

## Abbreviations

ACC	acetyl-CoA carboxylase
Acsl	acyl-CoA synthetase
AUC	area under the glycemic curve
B-H	Benjamini–Hochberg
Cish	cytokine-inducible SH2 domain containing protein
Cryab	crystallin alpha B
CVD	cardiovascular disease
DEG	differentially expressed genes
FDR	false discovery rate
GSH	glutathione
HMW adiponectin	high molecular weight adiponectin
hsCRP	high sensitivity C-reactive protein
HHTg	hereditary hypertriglyceridemic rat
KEGG	Kyoto Encyclopedia of Genes and Genomes
MCP-1	monocyte chemoattractant protein-1
MS	metabolic syndrome
NEFA	nonesterified fatty acids
OGTT	oral glucose tolerance test
OVX	ovariectomized HHTg rats
OVX+E2	ovariectomized HHTg rats receiving 17β-estradiol
PCA	principal component analysis
PMAT	perimetrial adipose tissue
PPARγ	peroxisome proliferator-activated receptor-γ
Scd-1	stearoyl-CoA desaturase-1
SEM	standard error of the mean
Serpine1	serpin family E member 1
SHAM	sham-operated HHTg rats
qPCR	quantitative real-time PCR
TG	triglycerides
