# Supplementary material for: The Effect of Ovariectomy and Estradiol Substitution on the Metabolic Parameters and Transcriptomic Profile of Adipose Tissue in a Prediabetic Model
Source: Antioxidants (Basel). 2024 May 21;13(6):627. doi: 10.3390/antiox13060627 (PMC11200657; doi:10.3390/antiox13060627)
Supplement: Supplementary file 1 [file antioxidants-13-00627-s001.zip › Supplementary Figure 1.pdf]

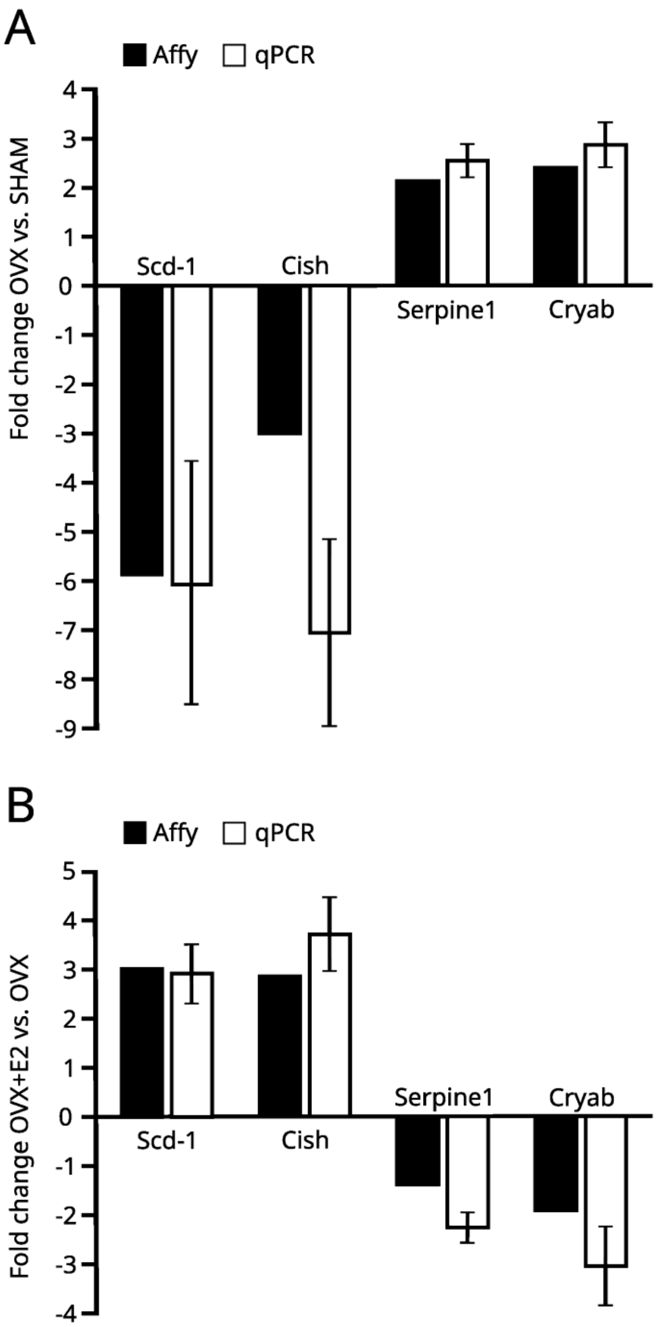

Supplementary Figure S1. Quantitative real-time PCR (qPCR) validation of selected significantly upregulated and downregulated genes in: Panel A: ovariectomized (OVX) vs. sham-operated (SHAM) female HHTg rats; Panel B: ovariectomized HHTg rats treated with 17β-estradiol (OVX+E2) vs. ovariectomized female HHTg rats. Fold changes are displayed based on microarray (Affy, Rat Gene 2.1 ST Array, black bars) and qPCR (white bars, data expressed as mean ± SEM), n=6 for each group. *Scd-1*: stearoyl-CoA-desaturase-1; *Cish*: cytokine inducible SH2 containing protein; *Serpine1*: serpin family E member 1 ; *Cryab*: crystallin alpha B.
