# Supplementary material for: The Effect of Ovariectomy and Estradiol Substitution on the Metabolic Parameters and Transcriptomic Profile of Adipose Tissue in a Prediabetic Model
Source: Antioxidants (Basel). 2024 May 21;13(6):627. doi: 10.3390/antiox13060627 (PMC11200657; doi:10.3390/antiox13060627)
Supplement: Supplementary file 1 [file antioxidants-13-00627-s001.zip › Supplementary Figure 2_OVX vs. SHAM.pdf]

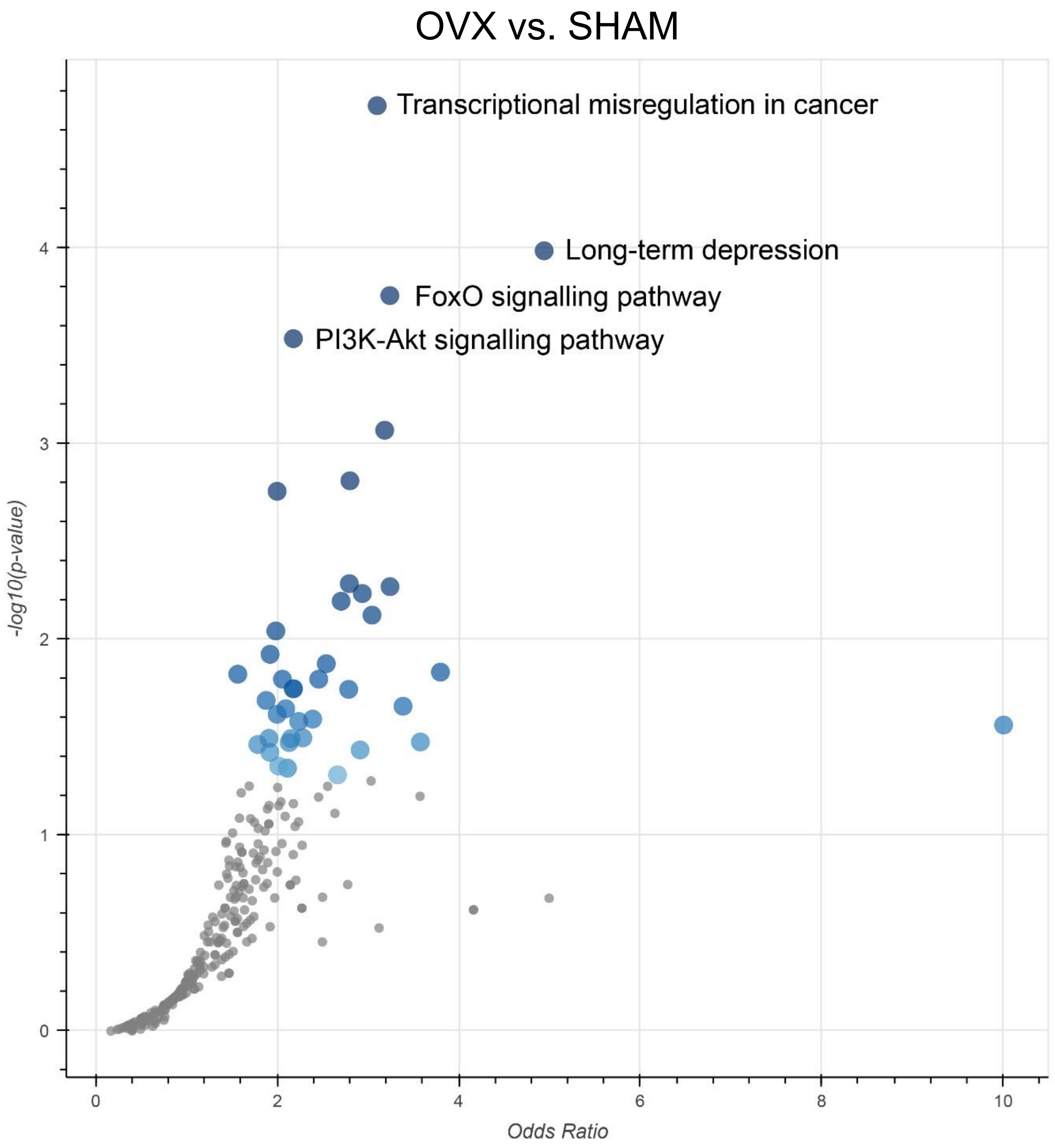

Supplementary Figure S2. Volcano plot of terms from the KEGG Pathway database. Each point represents a single term, plotted by the corresponding odds ratio (x-position) and -log10(p-value) (y-position) from the enrichment results of the DEGs set of OVX vs. SHAM comparison. The larger and darker-colored the point, the more significantly enriched the input gene set is for the term. The name labels are provided only for the pathways passing the adjusted significance level (Benjamini-Hochberg; $q < 0.05$).
