# Supplementary material for: The Effect of Ovariectomy and Estradiol Substitution on the Metabolic Parameters and Transcriptomic Profile of Adipose Tissue in a Prediabetic Model
Source: Antioxidants (Basel). 2024 May 21;13(6):627. doi: 10.3390/antiox13060627 (PMC11200657; doi:10.3390/antiox13060627)
Supplement: Supplementary file 1 [file antioxidants-13-00627-s001.zip › Supplementary Figure 3_OVX+E2 vs. SHAM.pdf]

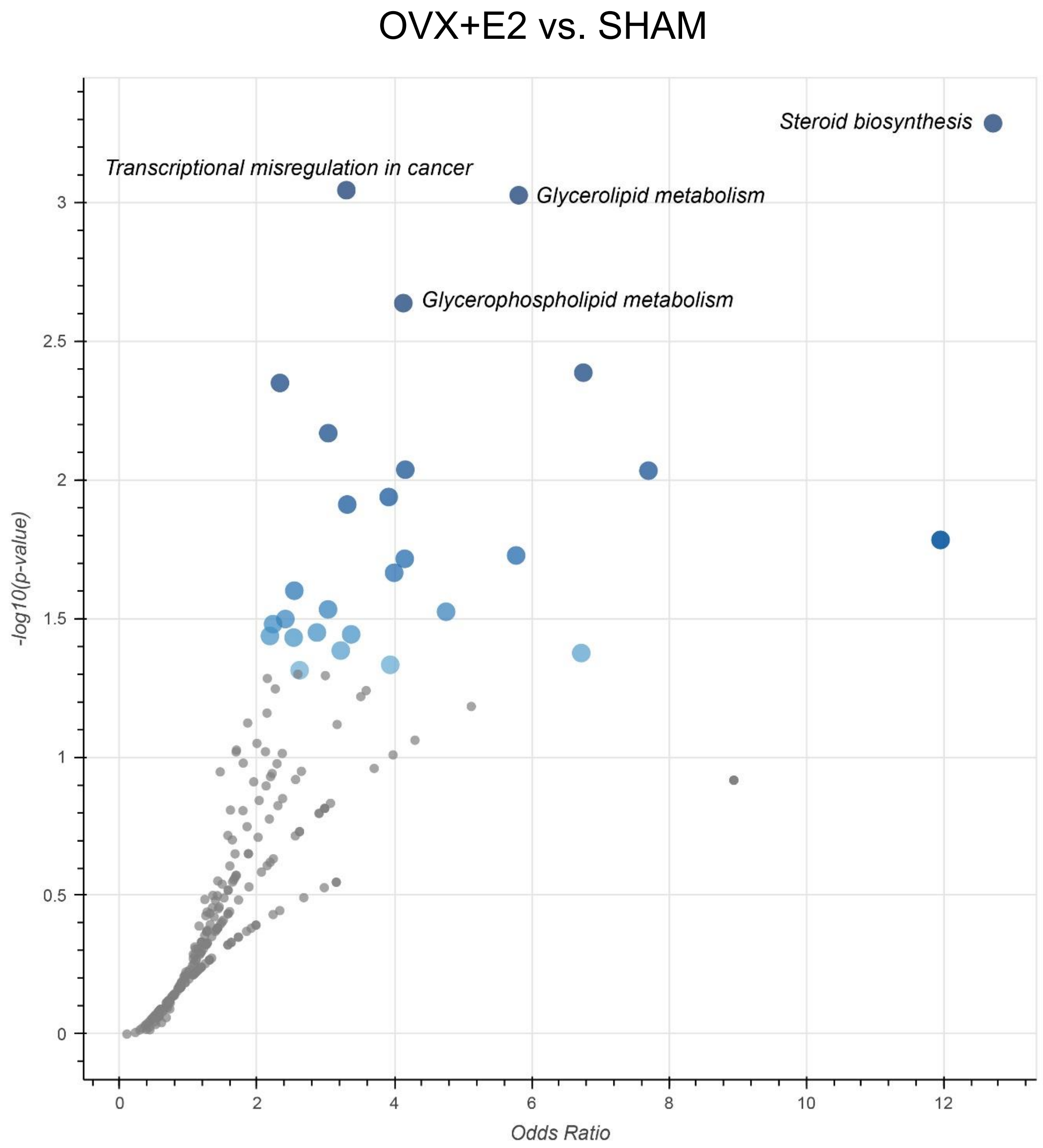

Supplementary Figure S3. Volcano plot of terms from the KEGG Pathway database. Each point represents a single term, plotted by the corresponding odds ratio (x-position) and -log10(p-value) (y-position) from the enrichment results of the DEGs set of OVX+E2 vs. SHAM comparison. The larger and darker-colored the point, the more significantly enriched the input gene set is for the term. None of the pathways surpassed the significance threshold after correction for multiple testing (Benjamini-Hochberg; $q < 0.05$). The name labels are provided for the top nominally significant pathways.
