# Supplementary material for: The Effect of Ovariectomy and Estradiol Substitution on the Metabolic Parameters and Transcriptomic Profile of Adipose Tissue in a Prediabetic Model
Source: Antioxidants (Basel). 2024 May 21;13(6):627. doi: 10.3390/antiox13060627 (PMC11200657; doi:10.3390/antiox13060627)
Supplement: Supplementary file 1 [file antioxidants-13-00627-s001.zip › Supplementary Figure 4.pdf]

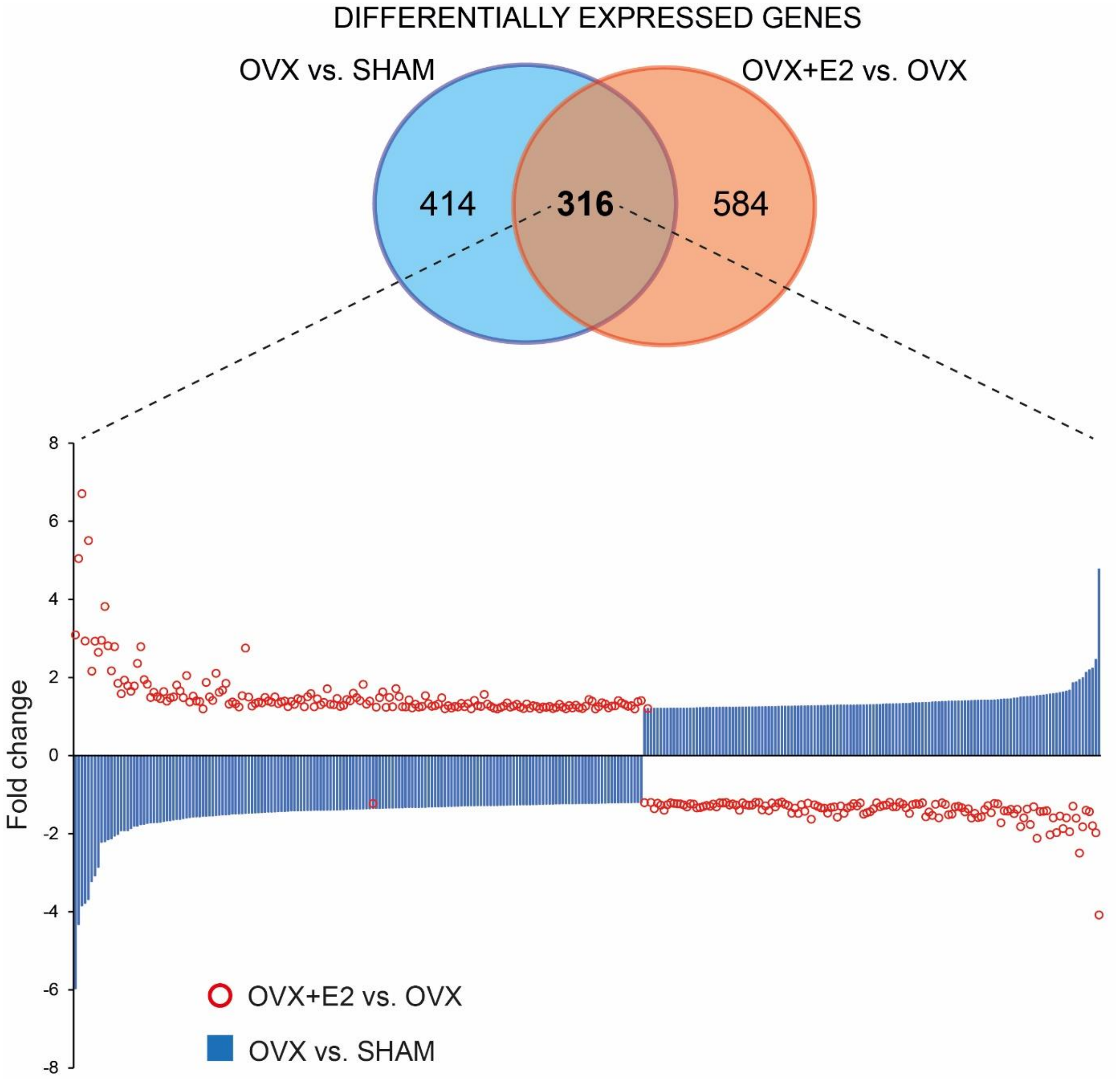


**Supplementary Figure S4.** Detailed comparison of 316 differentially expressed genes overlapping between datasets comparing the expression in ovariectomized rats vs. sham-operated HHTg female rats and (OVX vs. SHAM; fold change indicated as blue bars) and in OVX substituted with 17β-estradiol vs. OVX (OVX+E2 vs. OVX; fold change indicated as red circles).
